# Spirituality and religion and the role in improving teaching approaches to diversity and inclusion in the nursing and midwifery curriculum: an explanatory sequential multi-methods study

**DOI:** 10.1186/s12912-025-04193-4

**Published:** 2025-12-25

**Authors:** Barry Quinn, Debbie Duncan, Jeffrey R. Hanna

**Affiliations:** 1 School of Nursing and Midwifery, Queen’s University Belfast, Lisburn Road, Belfast, UK; 2 City St Georges, University of London, Northampton Square, London, UK; 3 School of Nursing and Paramedic Science, Ulster University, York Street, Belfast, UK; 4 South Eastern Health and Social Care Trust, Ulster Hospital, Dundonald, UK

**Keywords:** Spirituality, Religion, Culture, Education, Beliefs, Diversity, Inclusion, Nurses, Midwives, Students

## Abstract

**Background:**

Spirituality and religion play an important role in many people’s lives. While healthcare professionals support people from a diverse range of backgrounds, cultures and belief systems, these dimensions are often missing from assessments and care plans. Using the lens of nursing and midwifery students and academic teaching staff, this study sought to explore how the concepts of spirituality and religion could be better incorporated into nursing and midwifery teaching programmes, while acting as a possible conduit for exploring the richness of diversity and inclusion.

**Methods:**

An explanatory sequential multi-methods study, to include an online survey (*n* = 114 responses) and focus groups (*n* = 11 participants). Quantitative data were analysed using descriptive statistics and qualitative data were analysed using reflexive thematic analysis. Integration of the quantitative and qualitative data was achieved through a pillar integration process.

**Results:**

The concept of spirituality was viewed as predominately positive, something personal to individuals and linked to how people make sense of their place in the world. Religion was seen as a connectedness to a community with common beliefs and a shared identity. However, the rules and regulations associated with religion, were perceived by some respondents as leading to intolerance and the exclusion of others. Overall, participants believed that greater awareness of spirituality and religion could help people to be more aware and to be more welcoming of diversity leading to greater inclusion. Participants believed that these concepts should be included in teaching programmes and integrated with clinical practice.

**Conclusions:**

Students and clinical practitioners should be encouraged to increase their knowledge and awareness towards spiritual and religious issues. This awareness may begin with students and clinical practitioners reflecting on their own beliefs and values enabling them to be more sensitive to and respond to the beliefs and values of others. Insights gained by this study may be valuable to healthcare educators and policymakers highlighting the need for greater awareness of spirituality and religion in health and social care training.

**Clinical trial number:**

N/A.

**Supplementary Information:**

The online version contains supplementary material available at 10.1186/s12912-025-04193-4.

## Background

Despite the ongoing focus on person-centred care [1], the role of spirituality continues to be missing from many healthcare assessments and care plans. Austin and colleagues [2] highlight that when healthcare professionals recognise and address the spiritual needs of those who are ill, the quality of care delivered is enriched. Alongside this reality, health and social care professionals work within what can be described as a post secular world,^3^ engaging in supporting people from a diverse range of backgrounds, cultures and belief systems [4]. This requires clinicians to be welcoming and mindfully inclusive of each individual and their family members, with their unique lived experience and world view [5]. 

The term ‘spirit’ originates from the Latin ‘spiritus’, meaning ‘breath’ or ‘that which brings life’.^6^ Therefore, spirituality may be defined as anything that breathes meaning into a person’s life, giving meaning to their existence. Buber [7] contended that each person engages with life in and through their relationships with other people, life events, and beliefs and values. Frankl [8] asserted that every human being is driven by a conscious or unconscious desire to find a meaning in life; a belief in someone, or something that is worth striving for. In a similar way to Buber [7], Frankl [8] maintained that a core part of this meaning is found through relationships with family and friends, through engaging with life, and through belief in a higher being or beings. For Buber [7] and Frankl [8], these key relationships and the continuing search for meaning are present in every person and are at the heart of what it means to be spiritual [6]. 

More recently, Cobb and colleagues [9] referred to spirituality as something that gives meaning and purpose to one’s life. While Johnson and Walker [10] described spirituality as a universal search for meaning and purpose in life, Lalani [11] refers to spirituality as those often-hidden values which are closely linked to a person’s beliefs, their culture and how they view the world. This insights suggest that spirituality is part of the essence of who each person is as a human being and is therefore integral to health and social care practice.

Intertwined with spirituality, the concept of religion and the expression of a diverse range of religious practices and religious cultures, play an important and meaningful role in this broader notion of spirituality and how this might be expressed within health and social care [3, 12]. Through formal and informal religious practices, beliefs and rituals, many people living with illness find great comfort, solace and meaning through their religion and being connected within a shared culture and community of fellow believers [13]. 

The World Health Organization (WHO) emphasises the importance of health care that moves beyond a bio-medical view of health and illness, to one that sees health as a resource for living which includes the notion of spirituality, while recognising the diversity that exists [14]. Similarly, this is recognised by the International Council of Nurses [15], who clearly state that nurses must engage with and respond to the patients’ spiritual needs, which may or may not include a religious component. Although recognised by the WHO as an essential domain of health and social care [14] and core to nursing care, [15] spiritual care is still one of the most neglected components of the healthcare system [11]. There is a requirement for healthcare training programmes to move towards a more inclusive and engaging curriculum, which recognises and includes the rich and global perspectives on health and illness that exists [16, 17]. This requires a more inclusive training programme that prepares practitioners, to respond to the world of diversity that exists [3]. 

Stanford [16] and Stonehouse [17] clearly state that every individual in health care has the right to be treated fairly and with dignity and respect, regardless of their age, gender, ethnic origin, sexual preference, economic status or religious beliefs (or non-beliefs). This aligns to the principles and values set out by the WHO [18]. This requires all health and social care professionals to be prepared, and willing to engage with the rich and diverse world in which health and social care is delivered [19] and to recognise the need for greater inclusion [5]. 

The notion of equity is based on fairness [16, 17]. In health and social care this means equal opportunities for every person in need, [19] requiring professionals to recognise and respond to people’s diverse needs and values [19]. Inclusion is based on the belief that all people in society are entitled to share in society’s benefits and resources [24]. In relation to health and social care, this means that all people should feel welcome to live as part of their communities, and benefit from the facilities that some people in society may take for granted [20]. Inclusivity requires recognising those who experience being marginalised and, in some cases, ostracised from some health and social care services and support [19, 20] Diversity requires health and social care professionals to both recognise and to celebrate that differences do exist, which are not seen as a threat, but as enriching society as a whole [20]. Diversity also recognises the common needs that unite people in society, including the need for good health and social care support [21]. 

Building on previous research [22] and using the lens of nursing and midwifery students and academic nursing and midwifery teaching and research staff, the aim of this study was to explore how the concepts of spirituality and religion could be better incorporated into nursing and midwifery teaching programmes, while acting as a possible conduit for exploring the richness of diversity and inclusion. The study objectives were to investigate staff and students’:

1. Individual beliefs regarding spirituality and/or religion,
2. Perceptions of spiritualty and religion amongst nursing and midwifery students and academic staff, and how this might impact on engaging with diverse beliefs and values.
3. Perceptions of how best spirituality or religion can be incorporated into nursing and midwifery teaching programmes.

## Methods

This was an explanatory sequential multi-methods study [23]. An online survey was completed by nursing and midwifery students and academic staff to explore their views, beliefs and values regarding spirituality, religion and diversity and the relevance and importance to clinical practice and the curricula. Online focus groups were then completed after the survey data was analysed to expand on these findings. Adopting a multi-methods design was considered appropriate to enable complementarity and expansion of data, with both quantitative and qualitative components given equal weighting for this study [24]. 

### Setting

This was an online study conducted between December 2022 and March 2023 within one higher education setting in the UK. Eligible individuals were invited via email from a relevant department administrator to take part in the study.

### Participants

Individuals were relevant for inclusion if they were enrolled on a pre-registration or post-registration nursing and midwifery programme at the institution at the time of the study. This included individuals who were enrolled on any postgraduate research pathways, such as a PhD or MPhil. This wide sample was chosen as they either provide direct care and support to people who access health and social care perspectives (pre-and-post registration students), or they may have conducted research with the same populations and may have additional insights to offer. Academic members of staff with clinical, educational or research responsibilities related to nursing and midwifery at the same institution were also eligible for inclusion to the study. This sample was chosen as they either provide teaching within the institution, or they may have conducted research with students or people who access health and social care services regarding spirituality and may have additional insights to offer.

### Sampling

Convenience sampling techniques were used to identify eligible individuals. It was the authors intention to obtain a minimum of 80 survey responses to get a sense of the topic.

### Recruitment

#### Survey

An invitation email was circulated to eligible nursing and midwifery students and academic staff within the institution in December, week 1, 2022. The email provided individuals with a participant information sheet informing them about the purpose of the study, what participation would involve, the risks of participation, how the data would be used in the study and who to contact (the third author [JRH]) regarding any queries or concerns about the study before participation. The email also provided individuals with a link to an online consent form. Completion of the online consent form provided participants with a separate link to the survey. A further email invitation was sent to aid accrual to the study in January, week 1, 2023. The online survey was closed on February, week 1, 2023.

#### Focus groups

Individuals were invited to provide their email address in a separate form post-completion of the survey if they were interested in taking part in a focus group related to the survey and topic of spirituality, religion and diversity in nursing and midwifery. Individuals that expressed an interest to take part in a focus group were contacted via email on March, week 2, 2023 by the third author (JRH), and were provided with a participant information sheet detailing what participation in a focus group would involve, possible risks and benefits, their rights, privacy and confidentiality and whom to contact the third author [JRH] if they wished to take part or have other queries about the study. Eligible individuals confirmed their willingness to take part in focus group by return of email to the third author [JRH]. Written consent was obtained prior to the focus group, and verbal consent at the beginning of the focus group recording. Of note, no follow-up emails were sent to aid accrual to the focus groups to prevent individuals who may have been known to the research team from feeling pressurised to participate.

### Data collection

#### Survey

Following a systematic search of electronic databases and hand-searching of relevant journals by members of the research team (BQ, JRH), no validated instruments were deemed relevant for this study. The survey was developed by the research team and refined following feedback from a panel of peer-reviewers (*n* = 5) with relevant subject and methodological expertise. Participants completed single-items questions on a five-point Likert scale ranging from 1 (strongly disagree) to 5 (strongly agree) that were specific to the study aims and objectives (supplementary file 1). Open-ended text boxes were provided for participants to provide additional responses as appropriate. The survey deliberately did not offer any definitions of spirituality or religion in order to allow the emerging findings to be participant driven. Relevant socio-demographic questions were captured at the end of the survey. Of note, questions were not mandatory in completion of the survey.

#### Focus groups

Of the 21 individuals that expressed an interest from the survey, only 11 replied to the email invitation. Consequently, all individuals expressing an interest to participation were included in a focus group. Given no follow-up took place, it is unclear as to the reasons for non-participation for those that initially expressed interest. The eleven individuals were randomly allocated to one of two focus groups. A topic guide with open-ended questions was developed, informed by the literature, study aims and objectives and the findings from the survey, alongside the research team (BQ, JRH, DD) to guide the focus group conversation. The guide was iteratively modified after the first focus group to ensure follow-up with categories in the second focus group (Table 1). The focus groups were facilitated by the third author (JRH) with the first author (BQ) present as a note-taker and to be available should any participant appear to show signs of becoming upset or distressed. Both JRH (he/him) and BQ (he/him) are registered nurses and academics with a wealth of experience in qualitative interviewing on sensitive topics. The researchers were known to some of the participants in the focus groups, and some of the participants were known to each other. Participants were informed that personal information collected in the study would only be seen by the research team, and that any direct quotes would be anonymised. Participants were asked to provide the same reassurances that what was shared within the group would remain confidential. Focus groups were conducted on MS Teams on April, week 3, 2023, lasting 47 and 53 min respectively with the recording audio and visually recorded.

**Table 1:** Topic guide

**Sample of topics to guide the focus group informed by the literature, study aims and objectives, findings from the survey and the research team**
- Exploration of perceptions and beliefs about spirituality. - Exploration of perceptions and beliefs about religion. - Exploration of perceptions about whether spirituality/religion supports people to be more open and understanding of other people’s culture, beliefs and values. - Exploration about how spirituality and religion could be (if required) incorporated better to nursing and midwifery curricula.
**Sample of additional topics explored in the second focus group**
- Challenges and opportunities to talking about spirituality and religion in the classroom within divided communities. - Challenges and opportunities to talking about spirituality and religion in a healthcare context within divided communities.

### Data analysis

The integration of the quantitative and qualitative data was achieved through a pillar integration process [25, 26]. This involved four stages: listing, matching, checking and pillar building [26]. 

#### Quantitative

Quantitative data from the survey were exported to SPSS Statistics v.29. Descriptive statistics were used to analyse the single-item and socio-demographic questions by the third author (JRH).

#### Qualitative

Recordings from the focus groups were transcribed verbatim by the first author (BQ) and verified by the third author (JRH). Open-ended responses from the survey and the focus group data were analysed using the principles of reflective thematic analysis [27–29]. Initially, the first author (BQ) read and re-read the transcripts and open-ended responses to get a sense of the data. Then, BQ completed initial coding of the transcripts by marking similar words and phrases within the transcripts. This process was supported using mind maps [28]. Codes were reviewed and refined through discussion with JRH. Following this, BQ identified where some of the codes merged together, identifying four themes. Themes were discussed and refined through critical discussion with all co-authors.

#### Integration of quantitative and qualitative data

The integration of quantitative and qualitative data was completed by the third author (JRH). First, the data were arranged into two columns: quantitative and qualitative. A description of the interpretation of findings were listed beside each data item (listing). The quantitative and qualitative data were then aligned with each other in joint display as appropriate (matching). The data was then reviewed and refined independently by all co-authors to ensure accuracy of the interpretation (checking). There were no conflicts in this process. The insights from this process were conceptualised as pillars that formed the central column of the joint display (pillar building). Overall, two pillars were identified. The pillar integration process can be viewed in the Supplementary Fig. 1.

### Ethical considerations

Participants were informed that they did not have to take part, and non-participation would not impact the relationship they had with the University. Participants were informed of their right to withdraw from the online survey or focus groups at any point without negative impact. However, participants were informed that any data already collected during the survey or online focus groups prior to their withdrawal would be retained for the study. Pseudonyms have been applied to the quotes to protect the anonymity of participants. Focus group recordings were destroyed following transcription. Completed surveys and anonymised transcripts are stored on secure database with a protected password protect accessible by the research team. Data protection procedures are in place to destroy of all data in accordance with General Data Protection Regulation and the Data Protection Act 2018. Ethical approval was granted through a University Ethics Committee.

## Results

### Survey

A total of 114 survey responses were recorded. Of these, 85 (74.6%) were nursing and midwifery students (67 were completing a pre-registration programme, and 18 were completing a post-registration programme). There was a total of 29 academic staff members that completed the survey (25.4%). Of the combined sample, 53/114 (46.5%) were aged between 18 and 34 years, 51/114 (44.7%) were aged 35–54 years, and 10/114 (8.8%) were aged 55 and over. Of these, 15/114 (13.2%) identified as male and 99/114 (86.8%) identified as female. 71.9% (82/114) of the sample identified as Christian, 2.6% (3/114) as Buddhist, 5.3% (6/114) as Muslim, and 20.2% (23/114) as having no religion.

### Focus groups

A total of eleven individuals took part in one of two focus groups. Of these, seven were either a pre-registration student nurse or midwife, one was a post-registration student nurse, and three were academic members of staff with significant responsibilities in either education (*n* = 2) or research (*n* = 1) relevant to nursing and midwifery.

Aligned to the pillar integration process, findings are presented under two pillars: (1) spirituality and religion may help people to be more aware of others’ beliefs and values, and (2) integrating spiritual and religious care in to person-centred care and nursing and midwifery education.

### Pillar One: Spirituality and religion May help people to be more aware of others’ beliefs values

Out of 108 respondents that answered the survey question, a total of 80 (74%) considered themselves as a spiritual person. With this, a total of 72% (n = 78/109) of the respondents ‘agreed’ or ‘strongly agreed’ that spirituality helped them to find meaning in life. Consistently within the open-text responses, survey respondents viewed spirituality as a way of making sense of life and oneself in the world, which might include a relationship with a God or a higher power.

*Spirituality is to believe in something bigger than me. It could be a God*,* it could be a way of life*,* it could be an experience*,* a comfort. For me*,* spirituality is recognising*,* believing*,* dreaming. (survey response)*

Similar to the survey responses, spiritualty was viewed by focus group participants as something that encompassed others, helping to connect people to one another and to the world. Also, spirituality was seen as something related to the individual person and a person’s beliefs and values. Furthermore, the concept of spirituality was seen by focus group participants as something ‘*positive and inclusive’* that was ‘*welcoming to all’*. This aligns with the 71% (78/110) of survey respondents that ‘agreed’ or ‘strongly agreed’ that spirituality supports people to be more open and understanding of other people’s culture, beliefs, values and choices. More often, spirituality was described by focus group participants as *‘more open and accepting’* than the concept of religion.

*It just seems more accepting because it’s kind of whatever you want. Like whatever it means to yourself*,* whereas religions… kind of more in a box*,* very organised and structured. Where spirituality*,* it seems to be more openness because spirituality is whatever it means to yourself. (Focus group 1*,* participant A)*

Identified in the survey and focus groups, spirituality was reported as something that contributes to the person’s well-being, mental health and a way of caring for self. In general, spirituality was perceived as something that brought benefits to peoples’ lives.

*Spirituality means to me a feeling of peace and connectivity*,* a calm*,* contented feeling that incorporates physical*,* emotional*,* intellectual*,* and social of health and well-being. (survey response)*

In contrast to spirituality, of the 104 survey respondents that answered the question, a total of 50 (48%) ‘agreed’ or ‘strongly agreed’ that they consider themselves to be a religious person. Alongside this, a total of 53% (55/103) ‘agreed’ or ‘strongly agreed’ that religion helps them to find meaning in life. Consistent with the open-text responses, survey respondents viewed religion as either an organised form of spirituality with a specific set of beliefs, a way of connecting with or having a relationship with God, or was considered to create problems within divided communities.

*Religion is a framework*,* set by an institution*,* with historical roots which instils and encourages certain beliefs about how to live with ourselves and each other. (survey response)*

Within the focus groups, participants felt that religion enables individuals to be part of a community which provides a sense of identity. With this, it was believed that being part of such a community was more often ‘*organised and structured*, in that certain rules, regulations and practices had to be followed and adhered to.

*It seems that religion just seems to have connotations of organised religion*,* joining a certain type of club or being or aligning yourself with a certain agreed set ways of thinking and being maybe considered part of that group (Focus group 2 – participant D)*

There was a shared consensus amongst focus groups participants that some of the aspects of *‘connectedness’* or *‘belonging’* that were associated with religion are becoming *‘lost’*, as it was perceived that more people are choosing not to be part of a religion. At the same time, it was considered that the rich sense of belonging to a community with rules, regulations and structure, could lead to other people not *‘fitting in’* and so were seen as not being part of such a community.

*Spirituality is just more inclusive*,* you know it doesn’t see the difference. The word religion seems to conjure up*,* you know*,* you’re either one or the other. (Focus Group 1*,* participant F)*

Identified in the survey and explored further in the focus groups, participants perceived religion as holding a certain level of intolerance towards others who did not follow ‘rules and regulations’ of a particular community. Religion was often viewed as being *‘prejudiced’* by focus group participants that was often linked to the idea of sectarianism because of complex political and cultural history within some societies. To that end, a total of 49% (50/102) of survey respondents ‘agreed’ or ‘strongly agreed’ that religion supports people to be more open and understanding of other people’s culture, beliefs, values and choices.

*I think people have the perception that if you have a religion*,* you’re against everybody else’s and that you won’t respect anybody else’s religion because you think that your beliefs are the right ones and that everybody else’s is wrong. (Focus group 1*,* participant C)*

While differences related to the meanings and practices of spirituality and religion were identified, focus group participants considered spirituality and religion as an important part of life for people, and are closely connected to key life events, such as birth and death. It was considered that during significant life events such as birth and death, spirituality and religion were seen as becoming increasingly important.

*At the moment*,* the moment of birth*,* I think no matter where you come from*,* if you’re tuned in and connected. Spiritual*,* there’s something absolutely holy and sacred there*,* no matter what background you come from*,* I would say. But*,* you know*,* that sort of atmosphere and environment can kind of be crushed by the medicalised clinical system. (Focus group 2 - participant B)*

### Pillar Two: Integrating spiritual and religious care to person-centred care and nursing and midwifery education

Although spirituality and religion were considered integral to providing person-centred care in routine practice, focus group participants felt this aspect of care was often ‘*ignored’* by nurses and midwives in clinical practice due to factors such as the perception of having a lack of time or *‘saying the* wrong thing’ that may cause offence. This was discussed particularly in relation to cultures with complex histories of political and religious tensions. There was also a recognition that many nurses are from a younger generation (< 60 years old) and that many of the people they care for (older generations > 60 years) may hold different attitudes to spiritualty and religion. Older generation patients were perceived by focus group participants as placing more value on religion and belonging to communities of faith. In contrast, nurses from a younger generation might be less inclined to engage with religious practices or communities and might favour a more individualised approach through spirituality.

*A lot of our student population have no faith. If you look at the census*,* that figure is rising (those stating they have no religious faith). But if you look at the population that we nurse there of an older generation and many of them do still have a faith. And so*,* some students were a bit like*,* I don’t have a faith. How do I talk to someone that does.’ Focus group 2 – participant A)*

However, it was believed that if nurses and midwives can ask people about personal issues such as *‘has your bowels opened*,* or have you passed urine yet’*, then nurses and midwives can talk to patients about their religious and spiritual needs. Focus group participants felt it would be helpful for the nursing and midwifery workforce to *‘move beyond’* the idea of completing a question on spirituality and religion as part of a patient assessment, or as one respondent said, ‘*form filling’*, to one of an ‘*open’* conversation. Predominately, participants described this ‘*open’* conversation as one where a nurse or midwife would invite the person to talk about what was important to them by asking open-ended questions such as, ‘*what is important to you?’* or *‘how can we support you?’*. While such questions might help to facilitate the conversation, it was seen as important to let the person be the guide, to lead the way.

*Whenever you are doing the nursing admission and there’s the box that says spirituality or religion*,* I can’t remember which phrased*,* but the bit that everyone ticks and says: ‘do you need to speak to anyone in the church or whatever?’ ‘No’*,* like great*,* and they move on and nobody discusses it more. Like*,* what’s important to you or*,* you know*,* is there somebody*,* I don’t mean even religious*,* but*,* you know*,* is there somebody you would like to talk to*,* or have you thought about the end of life or have you thought about*,* oh*,* I don’t know. (Focus group 1*,* participant D)*

To support the provision of spiritual and religious care in routine clinical practice, it was strongly reported in the survey responses and focus groups that spirituality and religion should be themes incorporated throughout nursing and midwifery education programmes and be embedded in each of the modules that made up the programme of studies. Indeed, of the 96 individuals that answered the survey question, a total of 73 (76%) respondents ‘agreed’ or ‘strongly agreed’ that spirituality and religion should be included in the curricula in nursing and midwifery teaching programmes.

*So*,* the more I think of it*,* spirituality and religion*,* if we don’t teach it*,* are we really*,* could we actually give dignified care. (Focus group 2 – participant E)*

A total of 85% (73/96) of survey respondents ‘agreed’ or ‘strongly agreed’ that there is a need for further teaching of spirituality and religion to help nurses and midwives respond to people’s culture, beliefs, values and choices. This was considered especially important as societies are becoming more multi-cultural and diverse as a result of *‘people moving about’.*

*The world is becoming increasingly cosmopolitan. It is essential that nurses*,* midwives and any other healthcare professionals are aware of and profoundly respect that other cultures may have a completely different set of values*,* may they be religious*,* spiritual*,* moral. How can you truly care for someone if you don’t understand where they come from and what they bring with them. (survey respondent)*

A diverse approach to teaching methods and strategies were identified across the survey responses and focus groups including lectures with invited speakers, case-studies, the use of simulation as well as digital resources. On occasion, some survey responses and focus group participants felt it would be helpful if nursing and midwifery curriculums acknowledge how best to care and support someone who may have different beliefs and values that differ from their own or the cultural context they work in.

*I don’t have an understanding of other religions. Like*,* I was raised in a Christian family and I don’t really have an idea about*,* say*,* Muslim culture or other kind of religions. Whereby in nursing*,* if someone was of another religion*,* I don’t think I would really have an awareness of how I can care for them in a way that’s catered towards their needs*,* towards the religion and things*,* and how I can support them because I don’t really have that knowledge. So that kind of holds me back quite a bit. (Focus group 1 – participant E)*

## Discussion

Findings identified the concept of spirituality as something personal to individuals that relates to how one makes sense of their place in the world, alongside a connection with others, God, and the world around them. Religion was seen as connectedness to a community, culture or organisation that share in a faith, common beliefs and a shared identity. There were some concerns that rules and regulations linked to religions could lead to the exclusion of others as well as being less inclusive than spirituality. Alongside this, there were some concerns about religion being perceived as being intolerant, with a potential for prejudice and sectarianism. While spiritualty was perceived as positive and largely inclusive, religion was deemed as both positive and negative leading to some people feeling excluded. Nevertheless, most participants believed spirituality and religion should be more integrated into nursing and midwifery teaching programmes and within clinical practice, as a way of helping to promote better understanding, and a more inclusive approach to health and social care.

In this study, spirituality and religion were considered to help people find meaning in their lives. Literature highlights that spirituality can support people who are ill to make some sense of their illness or disease and have a positive impact on their healing process and well-being [30, 31] Studies, including the current study, show that people living with illness or in need of help often desire spiritual care, and nurses, midwives and healthcare professionals express a willingness to provide it. [32, 33] Wattis, Curran and Rogers [34], similar to the earlier and seminal works of Frankl [8] and Buber [7] suggest that concepts of meaning, purpose, hope, connectedness and values are central to the understanding of what spirituality means for each person, including those living with illness and in need of nursing care.

It has been reported that including spirituality within nursing education has a vital role in promoting spiritual awareness and supporting competence in this area of practice [35]. Alongside this, providing spiritual care education in the nursing curriculum can help students to enhance their self-efficacy to the provision of spiritual care [36]. Other studies have highlighted that nurses who deliver spiritual care recognise the value of this aspect of their role, feeling they are helping to promote the wellbeing of the people they care for [37, 38] However, despite the recognition of spirituality and religion as important components of health and wellbeing, [39, 40] literature consistently highlights that there is still a lack of focus on spirituality and religion in clinical practice and within nursing education [41]. It could be argued that concerns about the negative connotations about the role of religion in society can lead to avoidance of the topic by nurses and midwives in clinical practice. Similar findings have been reported in the literature [42]. 

Studies have shown that nurses continue to experience uncertainty around their role in spiritual care, necessitating reassurance, guidance and training [43, 44] In order to address these concerns, it is important to recognise the context in which health and social care is delivered [45]. Taylor suggests that there is a need to recognise and better understand the notion of ‘secularisation’ which cannot be seen as the absence or the negation of religion or spiritualty [46]. Rather, religion and spirituality should be reflected in the growing diversity of people’s beliefs and values, alongside, the perceived privatisation of these elements of people’s lives [46]. Concerns such as causing offense to another can lead to these important dimensions of life not being addressed or discussed. [32, 33, 46] There is a need for post-secular negotiation in clinical practice, which includes the concept of ‘existential communication’ whereby the lived experiences of the person in need, and the professional offering support are recognised, respected and valued, without either person’s beliefs and values being compromised. [3] Spiritual care can be integrated into secular healthcare systems, encouraging nurses and midwives to navigate spiritual and existential themes with sensitivity and openness, without compromising their own professional boundaries [3, 32, 33]. 

A further obstacle to the provision of spiritual care in clinical practice is the reality that many nurses, midwives and other healthcare professionals may not be aware of their own beliefs and values [45]. Wang and colleagues [48] suggest that nurses need to be aware of their own spirituality, beliefs and values in order to be able to deliver effective person-centred care. Ultimately competent spiritual and religious care must remain central to providing holistic care in an equitable manner [47–49] If nurses and midwives are supported to be more aware of spirituality and religion and the role they play within the world, they might be better placed to recognise and engage with the diversity that exists within health and social care practice, including the diverse beliefs and values of those they care for and work alongside [48]. 

One approach to address the provision of spiritual care in clinical practice is to influence future generations through the curriculum of all pre-registration and post-registration nursing and midwifery programmes. There is a requirement to educate and equip the current and future nursing and midwifery workforce with the language, skills and institutional support to engage with a person’s spiritual needs in a meaningful way [50]. Patrick and Chan [51] suggest that increasing spiritual and religious literacy will support students to develop the skills needed to be more inclusive and to deliver justice and equity in clinical practice. It is important for nursing and midwifery educators to create a safe learning environment where students are supported to critically examine the richness of their own beliefs and values, and to explore the richness and diversity of other’s beliefs and values and how these might be expressed through religion and spirituality [32, 42–44]. As a result, students of nursing and midwifery may be better able to engage with these concepts and act in turn as a conduit to appreciating the beliefs and values of others in clinical practice [33, 43]. Rogers and Wattis [49] suggest that by exploring these diverse concepts in a safe space such as the classroom can help to reduce the confusion that may exist and help nurses and midwives in delivering spiritual and religious care. Timmins and Caldeira [52] suggest that because spirituality and religion will mean different things to different people, this results in both a rich and complex aspect of person-centred care. However, rather than seeing this as something daunting, engaging in these aspects of care can enrich practice and the role of the nurse and midwife. These advancements are to be welcomed and embraced so that spirituality and religion can continue to be recognised and valued as an integral component of person-centred care that recognises diversity and is more inclusive. [53]

### Strengths, limitations and future research

The study provided rich and insightful views of a group of students and educators who are currently engaged with nursing and midwifery practice on an often-neglected component of practice. The study has demonstrated how nursing and midwifery curriculums need to be more inclusive of these aspects of care and how this inclusive approach can support students and enrich clinical practice and care. Limitations included using a non-validated survey instrument which was developed specifically for the study. In the focus group, where some participants knew each other and others did not, this may have influenced the dynamics and openness of discussion and this could have influenced the findings. As the participant’s selfselected to take part in the study and the sample context is very narrow, it should be noted that participants may not be representative of broader populations, even within the local health and social care context. Participants were from one University in one part of the UK’; therefore results from this study need to be critically considered when applied to other University and health and social care settings. Further collaborative studies with several other Universities are required to explore in more depth the findings arising from this study. Further studies should explore the presence of spiritual and religious concepts and care within the current nursing and midwifery curriculums.

## Conclusion

The concepts of spirituality and religion have been for too long absent from many nursing and midwifery curriculums. What these concepts mean within a growing multi-cultural society with diverse beliefs, needs and values, necessitates that these concepts be a core part of any pre or post registration curriculum, preparing and developing those to work in health and social care. Nurses and midwives in all areas of clinical practice should be encouraged to increase their knowledge and awareness towards spiritual and religious issues. This knowledge and awareness may begin with students and staff within nursing and midwifery being encouraged to reflect on their own beliefs and values so that in turn they might be able to recognise, be more sensitive to and respond to the rich and diverse beliefs and values of others. The insights gained by this study will hopefully be of value to nursing and midwifery educators and policymakers in education highlighting the need for students and staff to be more self-aware about their own spirituality and religion to deliver true person-centred care.

## Supplementary Information

Below is the link to the electronic supplementary material.

Supplementary Material 1

Supplementary Material 2

## Data Availability

The data that supports the findings of this study are available upon reasonable request from the first author (BQ). The data are not publicly available due to privacy and ethical restrictions.
