# Supplementary material for: Spirituality and religion and the role in improving teaching approaches to diversity and inclusion in the nursing and midwifery curriculum: an explanatory sequential multi-methods study
Source: BMC Nurs. 2025 Dec 25;25:48. doi: 10.1186/s12912-025-04193-4 (PMC12805763; doi:10.1186/s12912-025-04193-4)
Supplement: Supplementary file 1 — Supplementary Material 1 [file 12912_2025_4193_MOESM1_ESM.docx]

**Survey Questions**

**1. Relationship to the x (please select the category you belong to)**

Student

Pre-registration nursing or midwifery student

Post-registration nursing or midwifery student

Postgraduate research (for example, PhD candidate)

Staff

Lecturer with the School of Nursing and Midwifery, QUB

Researcher with the School of Nursing and Midwifery, QUB

**2. Age (please select the category you belong to)**

18 – 24 years old

25 – 34 years old

35 – 44 years old

45 – 54 years old

55 – 64 years old

65 – 74 years old

75 years and above

**3. The Disability Discrimination Act (1995) defines a person as disabled if they have a physical or mental impairment, which has a substantial and long term effect (ie. has lasted or is expected to last at least 12 months) on the person’s ability to carry out day-to-day activities.**

**Do you consider yourself to have a disability for example in relation to your physical, hearing, visual or mental health) according to the terms given in the Disability Discrimination Act? (please tick one option)**

| Yes |  | No |  | Prefer not to say |  |
| --- | --- | --- | --- | --- | --- |

**4. Your ethnic group**

(These are based on the Census 2011 categories, and are listed alphabetically)

**Asian, Asian British, Asian English, Asian Scottish, Asian Welsh or Asian Irish**

| Indian |  | Pakistani |  | Bangladeshi |  |
| --- | --- | --- | --- | --- | --- |
| Other Asian Background |  |  |  |  |  |

**Black, Black British, Black English, Black Scottish, Black, Welsh or Black Irish**

| Caribbean |  | African |  | Other Black Background |  |
| --- | --- | --- | --- | --- | --- |

**Chinese, Chinese British, Chinese English, Chinese Scottish, Chinese Welsh or Chinese Irish**

| Chinese |  | Other Chinese Background |  |
| --- | --- | --- | --- |

**Mixed**

| White & Black African |  | White & Black Caribbean |  | White & Asian |  | Other Mixed Background |  |
| --- | --- | --- | --- | --- | --- | --- | --- |

**Other**

| Other |  | ***Please state:*** |
| --- | --- | --- |

| **Prefer not to say** |  |
| --- | --- |

**White**

| White-English |  | White-Welsh |  | White-British |  | White Non-European |  |
| --- | --- | --- | --- | --- | --- | --- | --- |
| White-Scottish |  | White-Irish |  | White-European |  | Other White background |  |

**5. Your gender**

| Male |  | Female |  | Prefer not to say |  |
| --- | --- | --- | --- | --- | --- |
| Non-binary |  |  |  |  |  |

**6. Your gender identity**

Do you identify to the gender assigned to you at birth.

| Yes |  | No |  | Prefer not to say |  |
| --- | --- | --- | --- | --- | --- |

**7. Your religion or belief**

| Buddhism |  | Judaism |  | Other (please specify below) |  |
| --- | --- | --- | --- | --- | --- |
| Christianity |  | Islam |  | Prefer not to say |  |
| Hinduism |  | No religion |  | Sikhism |  |

**8. Your sexual orientation**

| Bi-sexual |  | Heterosexual/straight |  | Gay |  |
| --- | --- | --- | --- | --- | --- |
| Lesbian |  | Other (specify if you wish) |  | Prefer not to say |  |

**Part 2: Spirituality focused questions**

**9. Using the space below, can you tell us in no more than 20 words what ‘spirituality’ means to you?**

[open ended text box]

**10. The questions below are about what spirituality mean to you. Please answer each statement using the scale provided, from strongly disagree to strongly agree.**

1. **I consider myself to be a spiritual person**

Strongly disagree, disagree, neither agree or disagree, agree, strongly agree

1. **My spirituality helps me find meaning in life**

Strongly disagree, disagree, neither agree or disagree, agree, strongly agree

1. **Spirituality supports people to be more open and understanding of other people's cultures, beliefs, values and choices**

Strongly disagree, disagree, neither agree or disagree, agree, strongly agree

**Part 3; Religion focused questions**

**11. Using the space below, can you tell us in no more than 20 words what ‘religion’ means to you?**

[open ended text box]

**12. The questions below are about what religion mean to you. Please answer each statement using the scale provided, from strongly disagree to strongly agree.**

1. **I consider myself to be a religious person**

Strongly disagree, disagree, neither agree or disagree, agree, strongly agree

1. **My religion helps me to find meaning in life**

Strongly disagree, disagree, neither agree or disagree, agree, strongly agree

1. **Religion supports people to be more open and understanding of other people's cultures, beliefs, values and choices**

Strongly disagree, disagree, neither agree or disagree, agree, strongly agree

**Part 4: Religion and spiritualty in nursing and midwifery teaching**

**13. The questions below are about religion and spirituality in nursing and midwifery teaching. Please answer the following questions using the scale provided from strongly disagree to strongly agree.**

1. **It is important for spirituality and religion to be included in the curricula in nursing and midwifery teaching programmes.**Strongly disagree, disagree, neither agree or disagree, agree, strongly agree
2. **There is a need for further teaching on spirituality and religion to help nurses and midwives respond to people’s cultural, beliefs. values and values**

Strongly disagree, disagree, neither agree or disagree, agree, strongly agree

**14. Is there any suggestions you have as to how spirituality or religion can be better incorporated to nursing and midwifery programmes?**

**15. Is there anything else you feel relevant to tell us about spirituality or religion?**
