# Supplementary material for: Spirituality and religion and the role in improving teaching approaches to diversity and inclusion in the nursing and midwifery curriculum: an explanatory sequential multi-methods study
Source: BMC Nurs. 2025 Dec 25;25:48. doi: 10.1186/s12912-025-04193-4 (PMC12805763; doi:10.1186/s12912-025-04193-4)
Supplement: Supplementary file 2 — Supplementary Material 2 [file 12912_2025_4193_MOESM2_ESM.docx]

**Figure 1: Pillar integration process**

| **Survey responses** | **Survey category interpretation** | **Pillar building themes** | **Focus group category interpretation** | **Focus group data** |
| --- | --- | --- | --- | --- |
| **Open-text: ‘Meaning of spirituality’**  ‘Spirituality is about being concerned for or caring for mine or someone else's spirit.’  ‘Spirituality for me is my belief in God, which effects my whole life including the things I do, say, think and feel.’  ‘Spirituality is how a person sees themselves in relation to the wider world and their environment. It is an awareness of something beyond our individual selves that connects us and gives meaning to life.’  ‘Spirituality is believing in a higher power, and something we look towards for comfort, hope and security. Not always necessarily a “god”.’  **Q. ‘I consider myself to be a spiritual person’ (n=108/114)**  Strongly disagree, n=5 (5%)  Disagree, n=5 (5%)  Neither agree not disagree, n=18 (17%)  Agree, n=41 (38%)  Strongly agree, n=39 (36%)  Missing, n=6  **Q. ‘My spirituality helps me find meaning in life’ (n=109/114)**  Strongly disagree, n=6 (6%)  Disagree, n=7 (6%)  Neither agree nor disagree, n=18 (17%)  Agree, n=37 (34%)  Strongly agree, n=41 (38%)  Missing, n=5  **Q. Spirituality supports people to be more open and understanding of other people’s culture, beliefs, values and choices (n=110/114)**  Strongly disagree, n=6 (5%)  Disagree, n=9 (8%)  Neither agree nor disagree, n=17 (15%)  Agree, n=47 (43%)  Strongly agree, n=31 (28%)  Missing, n=4  **Open-text: ‘Meaning of religion’**  ‘An organised group with a particular focus and similar goals, following rules, belief in a being that isn't human.’  ‘Religion to me is a specific set of beliefs based on a religion which is shared by the community. I think people can be spiritual but not religious’  ‘Organised and shared rituals and customs that are meaning to the participants that are linked to their shared belief that there is more to life than the physical dimension we see around us.’  ‘I think of big, organised Institutions, but on a personal level, it is my own private relationship with Allah in which I find peace, difficulty but a means to be resilient to life’s trials and tribulations.’  ‘Religion to me in Northern Ireland speaks about a divide in our community.’  **Q. ‘I consider myself to be a religious person’ (n=104/114)**  Strongly disagree, n=16 (15%)  Disagree, n=25 (24%)  Neither agree not disagree, n=13 (13%)  Agree, n=32 (31%)  Strongly disagree, n=18 (17%)  Missing, n=10  **Q. ‘My religion helps me to find meaning in life’ (n=103/114)**  Strongly disagree, n=16 (16%)  Disagree, n=16 (16%)  Neither agree nor disagree, n=16 (16%)  Agree, n=31 (30%)  Strongly agree, n=24 (23%)  Missing, n=11  **Q. ‘Religion supports people to be more open and understanding of other people’s culture, beliefs, values and choices’ (n=102/114)**  Strongly disagree, n=15 (15%)  Disagree, n=15 (15%)  Neither agree nor disagree, n=22 (22%)  Agree, n=32 (31%)  Strongly agree, n=18 (18%)  Missing, n=12 | Overall, open-text survey responses to the meaning of spirituality identified that it was a way of making sense of oneself; appreciating what you have in this world; a way of making sense of yourself within the world; your relationships with God; a belief in a higher power’ something that is individual to oneself; a connection with others  In general, relation was viewed as an organised form of spirituality with a specific set of beliefs; a way of connecting with and having a relationship with God; different from having a faith; considered to create problems within divided societies and communities | **Pillar 1: Spirituality and religion may help people to be more aware of others’ beliefs and values**  Overall, spirituality was seen as something that was more open and inclusive to others’ views, beliefs and values. In contrast, religion was viewed as something structured and organised that came with set rules and regulations. It was considered people are more spiritual than religious. Both spirituality and religion are important to people during key life events such as birth, illness and death. | Spirituality was perceived as being more focused on the individual and how the person interacted with the world and how they sought meaning in this context. Spirituality was less focused on rules and regulations and was seen as generally open and inclusive of all.  Religion was perceived as being linked to a community who shared similar values and beliefs, including a belief in a God.  Religion was linked to following certain rules and regulations, which could lead some people to feel excluded.  The importance of spiritualty and religion took on greater meaning at key points in someone’s life, including births and approaching death. | **Sub-theme one: Spirituality as more open and inclusive**  ‘Spirituality is just much more inclusive. You know, it doesn’t see difference. It's like the word religion seems to conjure up. You know, you are either one or the other’. Focus group 1, participant B  ‘We talk so much about it right and wrong. But I think all of these are valid and I think it's, you know, for me it's really important that when we're talking about spirituality with patients or even with our peers or colleagues, I think it's really important that there's that room and that grace, that there's not a one-size-fits-all and there's not an answer, but it what's similar?’. Focus group 1, participant E  **Sub-theme two: Religion as something structured and organised**  ‘I generally agree that the religion would have more of a like as you say guidance or framework’. Focus group 1, participant A  ‘Spirituality seemed to be about me more, and religion more about community. Religion is more about the community connecting together’. Focus group 2, participant A    **Sub-theme three: Religion being less open to others**  ‘Spirituality means more to people. You know, it’s so let me try and phrase this another way. I think it’s more because of where we live in Northern Ireland. I think religion does carry all of these potentially negative connotations and perhaps because of the community in the background that we’re from’. Focus group 1, participant D  **Sub-theme four: Religion and spirituality during significant life events**  ‘So, we did what we could, we had a room that we could cool right down. We had the child in the room that was cooled right down. And also when dad was going to pray before the sun came up, I just went round and sat with her (the child), we spent that time with her after she had passed so dad could go and do things, because the other thing that was very important to him (dad) personally, not necessarily from a religious point of view, was that she (the child) was never left’. Focus group 1, participant F.  ‘The old tradition of opening the window to let the spirit out, which I'm pretty sure every nurse in the ward does but would never have considered, that as spirituality. They just know that's nursing care. And I know there are places you can't open windows now, but we always did it. That is just what you do when somebody dies. And it's chatting to them (the person who has died) and you don't know if their spirit has left the body or not or you know, yes, they are dead. But how long do they linger (the deceased person)’. Focus group 2, participant D. |
| **Q. ‘Do you believe that spirituality and religion should be included in the curricula in nursing and midwifery teaching programmes?’ (n=96/114)**  Strongly disagree, n=4 (4%)  Disagree, n=9 (9%)  Neither agree nor disagree, n=10, (10%)  Agree, n=47 (49%)  Strongly agree, n=26, (27%)  Missing, n=18  **Q. Do you believe that there is a need for further teaching on spirituality and religion to help nurses and midwives respond to people’s culture, beliefs, values and choices? (n=96/114)**  Strongly disagree, n=4 (4%)  Disagree, n=7 (7%)  Neither agree nor disagree, n=4 (4%)  Agree, n=43 (45%)  Strongly agree , n=38 (40%)  Missing, n=18  **Open text: Religion and spirituality in nursing and midwifery curriculum**  **‘**Should be embedded more in our practice learning portfolio. Review school curriculum to see how many sessions actually focus on these subjects.’  ‘Spirituality is vital to life and is so neglected in Midwifery care. We need to move away from the dominant pathological model of practice and make space to be open to understanding both the worker and the family's experience of spirituality. It means collaboration with diverse disciplines, looking at history of birth, lived experience and cultural diversity.’  ‘Nurses should learn formally about all world religions. It should be included in the undergraduate programme and post registration. Nurses should continue to refresh and update their knowledge. Nurses are present for some of life’s biggest events: birth, illness and death - all of these are interpreted by individuals through the lens of their religion and spiritual beliefs. Nurses should be aware of this. | Overall, most respondents felt that spirituality and religion could be included within teaching programmes to ensure the provision of person-centred care. This was considered especially pertinent in ever-changing and more diverse communities and societies. | **Pillar two: Integrating spiritual and religious care to person-centred care and nursing and midwifery education.**  A number of challenges were identified to the provision of pastoral (spiritual and religious) care in routine clinical practice. Nonetheless, it was believed that nurses and midwives are integral to exploring the spiritual and religious needs of people to ensure the provision of person-centred care in routine practice. Educational and curriculum programmes were identified as integral to promoting spiritual care in practice, with a number of strategies identified. | Training should include supporting students to be open, welcoming and supportive of people whose identity might be different from their own.  There was a recognition that responding to spiritual and religious needs in clinical practice could be challenging and there was a concern over causing offence.  Training on spirituality and religion should be woven into each module/programme and not simply another topic or a simply another stand-alone session. | **Sub-theme one: Exploring the key priorities of patients**  ‘Whenever I ask that question, what is important to you? it might have been well, ‘I have a dog at home, and I don't know what I'm supposed to do with my dog’. And, it might not even have been anything religion or spirituality. And it just starts the conversation of, well, who have we got around that can help with the dog? And, you know, “If anything happens to you, what would you like to happen? What's the dog or, you know. It just started a conversation in a different way. And I think it just opened up and made the people maybe feel more at ease and talking about their problems’. Focus group 1, participant EB.  ‘I just have to be very, very careful and certainly not to impose my religious views on others. I'm very clear and I would always say to the students, when they put on the uniform and personally, they may be the greatest homophobe, the greatest, blah, blah, blah. But when they wear that uniform, they then have to be open and welcoming and it doesn't really matter, they need to challenge themselves’. Focus group 2, participant C.  **Sub-theme two: Challenges to integrating spirituality and religion to routine clinical care**  ‘But you know, religion, you could argue, has had so much of a negative impact in this country, particularly in divides. And, you know, this age-old war that we see particularly pertinent based on the anniversary that we're celebrating this week (referring to a recent celebration).’ Focus group 1, participant C  ‘You don't want to even address it, in case you insult somebody’. Focus group 2, participant E  **Sub-theme three: Integrating spirituality and religion to the curriculum**  ‘Disturbing, especially for students here, just straight from school and things. It's never really crossed their mind. Maybe that would be a big thing, you could be looking after a woman in labour and then, you know, the baby would be still-born. It's rare, but you know, it does happen’. Focus group 1, participant C  ‘Religion is too often, and spirituality, are associated with just the end of life, whereas the being present, the being there for people, to be able to understand, that the being present is in itself a spiritual thing, I think the spirituality should be included in every single module’. Focus group 2, participant B |
